# History of the International Union of Societies for Biomaterials Science and Engineering

**DOI:** 10.34133/bmr.0239

**Published:** 2026-02-06

**Authors:** Joachim Kohn, Insup Noh

**Affiliations:** ^1^ Rutgers University, Piscataway, NJ, USA.; ^2^ Department of Chemical and Biomolecular Engineering, Seoul National University of Science and Technology, Seoul 01811, Republic of Korea.

## Abstract

The International Union of Societies for Biomaterials Science and Engineering (IUSBSE) is a global organization in the field of biomaterials that brings together national and international biomaterials societies. It is dedicated to the advancement of not only biomaterials but also surgical implants, prosthetics, artificial organs, tissue engineering, drug delivery, and regenerative medicine. IUSBSE and the World Biomaterials Congress were established in 1996. This article highlights the efforts and contributions of IUSBSE’s past and current presidents from 1980 to 2024 in both academia and industry. The history of IUSBSE acknowledges its background, its role, and the lifetime contributions of its presidents in fostering international networks among students, researchers, and societies in biomaterials science, as well as advancing academic and industrial progress in support of human health.

## Introduction

The International Union of Societies for Biomaterials Science and Engineering (IUSBSE) is a global organization in the fields of biomaterials that brings together national and international societies dedicated to the advancement of biomaterials, implants, prosthetics, artificial organs, tissue engineering, and regenerative medicine. Their membership consists of biomaterials societies from all around the world. IUSBSE has 10 member societies: the Australasian Society for Biomaterials and Tissue Engineering, the Canadian Biomaterials Society, the Chinese Society for Biomaterials, the Chinese Taipei Society for Biomaterials and Controlled Release, the European Society for Biomaterials, the Society for Biomaterials and Artificial Organs India, the Japanese Society for Biomaterials, the Korean Society for Biomaterials (KSBM), the Latin American Society for Biomaterials and Artificial Organs, and the Society for Biomaterials (SFB-USA). These constituent societies are the direct members of IUSBSE, and their individual members are thus part of the broader international biomaterials science and engineering community fostered by the union. IUSBSE’s primary roles are organizing the World Biomaterials Congress (WBC) every 4 years and recognizing scientifically accomplished individuals through the Fellow of Biomaterials Science and Engineering (FBSE) designation. WBC is a highly prestigious and large-scale international academic conference in the field of biomaterials science. Held every 4 years since its establishment in Vienna, Austria (1980), it serves as a global platform for biomaterials experts to gather, share the latest research, and discuss diverse areas of biomaterials research and beyond.

To celebrate WBC2024’s success and consolidate the society members’ international connections for better biomaterials research and network, we here introduce the history of IUSBSE and express great thanks to all IUSBSE presidents for their lifetime contributions to the society and scientific developments. The chronical and important history of IUSBSE with their great contributions is acknowledged as follows.

## History of IUSBSE

### Part 1: The beginnings of IUSBSE from 1980 to 1996

The history of IUSBSE is part of the history of the field of biomaterials science. In the 1970s, Clemson University in South Carolina, USA, was widely regarded as the center of biomaterials research in the USA. In the 1980s, the world was relatively peaceful and increasingly prosperous. There was a strong commitment among early biomaterials scientists to work together and to find ways to formalize and foster international collaborations. This commitment was part of the strong personal relationships among leading biomaterials scientists in the USA, Europe, and Japan and was supported by the governments of the major industrialized nations.

In 1974, the US Society for Biomaterials (“SFB”) was formally established as the first national society representing the field of biomaterials research. In 1980, the First International Congress on Biomaterials was held in Vienna, Austria, with the participation of newly formed societies in France, Canada, Japan, and Belgium. This meeting is now widely regarded as the first WBC.

During this meeting, the International Liaison Committee of Societies for Biomaterials (ILC) was formed by the Canadian Biomaterials Society (CBS), European Society for Biomaterials (ESB), Japanese Society for Biomaterials (JSB), and Society for Biomaterials (SFB-USA). These societies also decided to organize a WBC every 4 years.

As described later, the ILC was the starting point for an unprecedented level of global collaboration, unique among other scientific disciplines.

Since 1980, the field of biomaterials science has experienced almost exponential growth. From 1980 to 2000, biomaterials science and engineering was the most pivotal contributor to innovation in global healthcare. The 2nd WBC was held in Washington (1984); the 3rd in Kyoto, Japan (1988); and the 4th in Berlin, Germany (1992). By this time, the 4 original biomaterials societies (CBS, ESB, JSB, and SFB) had been joined by 3 new societies as “observers” (China, India, and the Republic of Korea).

WBC1992 marked the beginning of honoring the most prominent biomaterials scientists as “Fellows of Biomaterials Science and Engineering”, abbreviated as FBSE. At the conclusion of WBC1996 (Toronto, Canada), the first 68 new Fellows were officially recognized. WBC1996 also marked the founding of IUSBSE. This decision was made at WBC1996 and formally adopted in 1997.

In writing the early history of IUSBSE, we have deliberately omitted the names and contributions of the founding fathers of the field of biomaterials science. It would have been a huge undertaking to recognize all of the truly exceptional scientists who shaped the field of biomaterials science from the 1970s to the 1990s. We would not have been able to do justice to their tremendous contributions to biomaterials science and global healthcare.

But there is one exception: We must mention Dr Andreas von Recum. Born and educated in Germany (Free University of Berlin), Dr von Recum became an expert in experimental surgery and trained at Colorado State University. He founded and directed 2 bioengineering departments, one at Clemson University and one at Ohio State University. In 1996, Dr von Recum served as the president of SFB-USA and was the driving force behind the formation of IUSBSE. He strongly believed in the need for international collaboration. It is possible that without Dr Andreas von Recum, IUSBSE would not have been founded in 1996. At that time, one of the authors (Joachim Kohn) served as the US delegate to the International Liaison Committee (ILC) and was able to witness the early days of IUSBSE firsthand.

In 1998, Dr von Recum registered IUSBSE in the USA as an official corporation. This registration has been maintained ever since.

### Part 2: The growth and activities of IUSBSE from 1996 to 2024

#### Chairman Andreas von Recum

**Fig. 1.**
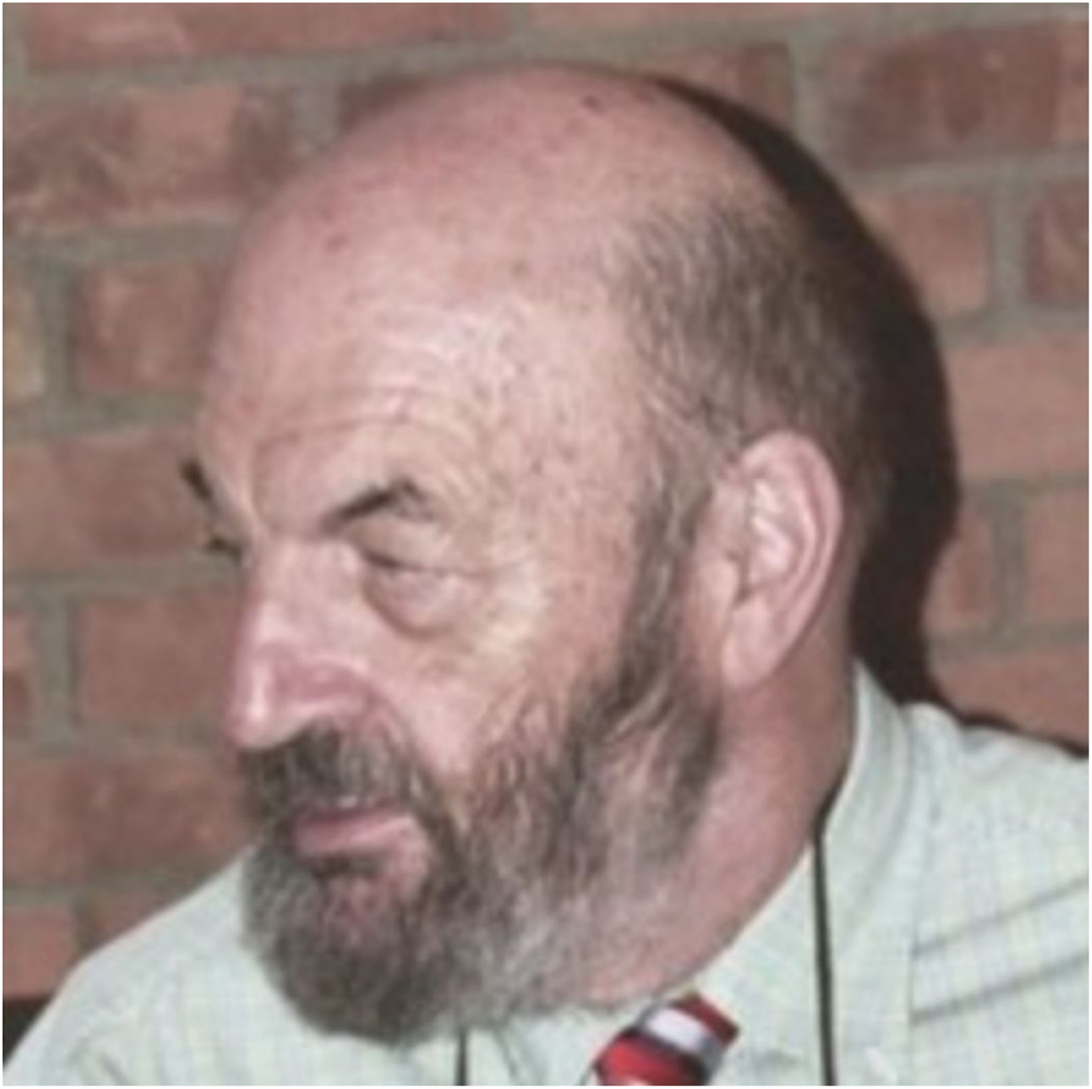
Dr Andreas von Recum (LinkedIn profile photo; reproduced with permission).

Professor von Recum was the first chairman of IUSBSE from 1996 to 2000 (Fig. 1). He led the preparation of the first guidelines for the activities and procedures of IUSBSE. Dr von Recum’s vision was to keep IUSBSE as a very limited organization. He correctly assumed that internal conflicts within this new and untested organization could be minimized by not having a budget and by defining a set of very limited activities: (a) once every 4 years, selecting the venue for the next WBC and (b) approving the nominations for Fellow status received from the participating biomaterials societies. During this time, Joachim Kohn served as one of the delegates representing SFB-USA. At that time, and ever since, IUSBSE was a very collegial organization. Each member society was represented by 2 delegates. Each of the delegates had one vote. All decisions were made by ballot. The delegates met in person, usually once a year during one of the major biomaterials meetings. Very often, the IUSBSE meetings were held in conjunction with the annual meetings of SFB-USA. Since IUSBSE had no budget or resources, one of the member societies was asked to provide a meeting room and a modest lunch for the participating delegates. All delegates paid for their own airfare and hotel accommodations. One of the key issues discussed during this time was the role of the Fellows (FBSEs). The delegates made an important decision: Unlike many other organizations that confer a passive Fellow status, the vision of IUSBSE was that Fellows should be active, serve as role models for younger scientists, and support the development of international collaborations among member societies.

Perhaps the most notable event during these early years was the rapid growth of tissue engineering and regenerative medicine. Prior to 1996, biomaterials research was dominated by large implants (such as hip and knee replacements), and most biomaterials in clinical use were metals, ceramics, and nondegradable polymers. The largest group of abstracts for WBC1992 was on wear particles in total hip and knee replacements. By 1996, however, the focus of WBC1996 had shifted to tissue engineering. In the following years, tissue engineers split off from the SFB to form the Tissue Engineering and Regenerative Medicine International Society (TERMIS). In retrospect, it would have been better if the founding fathers of tissue engineering had stayed with biomaterials. Instead, TERMIS became a major competitor to IUSBSE, organizing its own local, regional, and world congresses.

#### Chairman: Michael Lee, 2000 to 2004

**Fig. 2.**
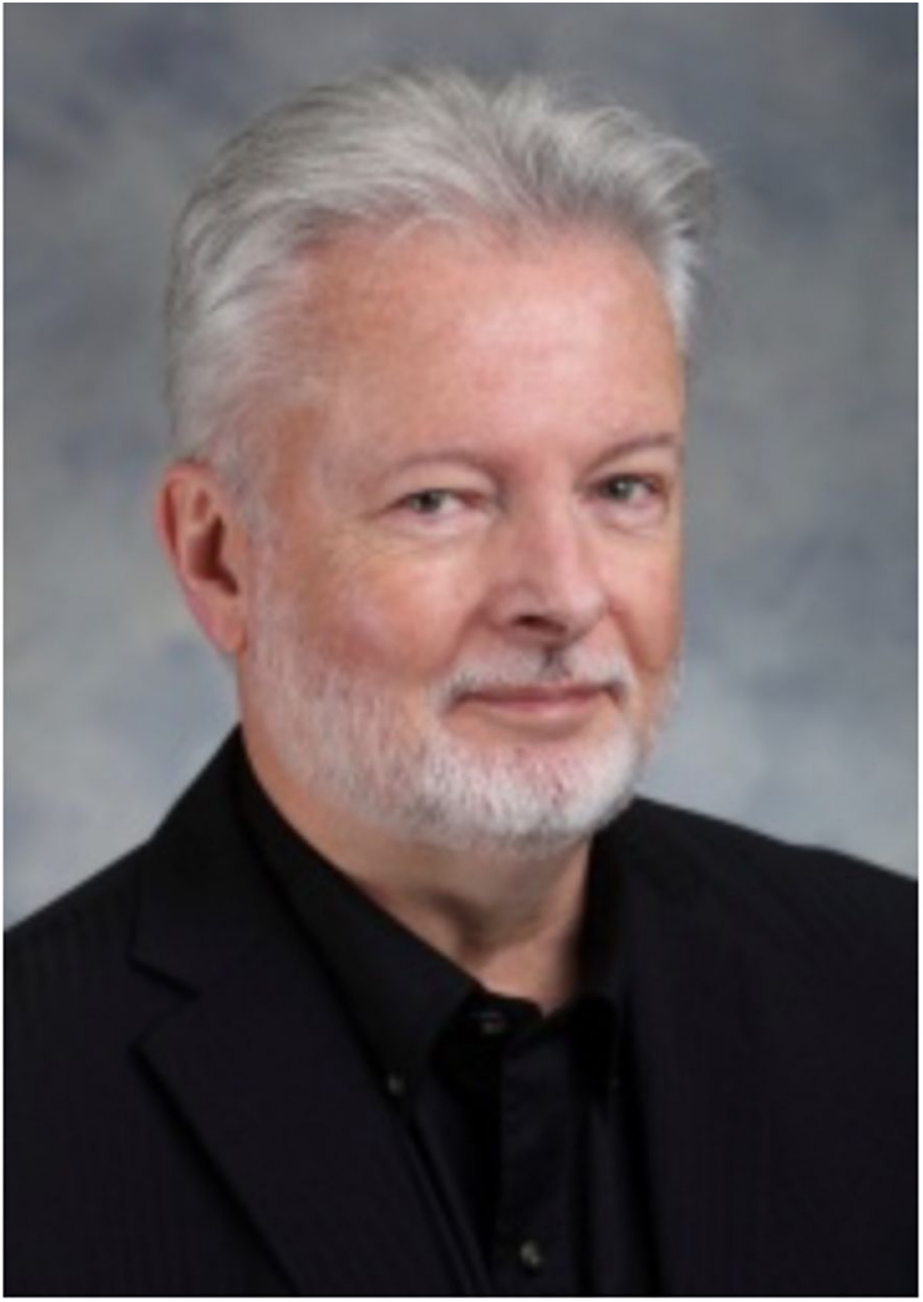
Dr Michael Lee, chair of the International Union of Societies for Biomaterials Science and Engineering (IUSBSE) from 2000 to 2004 (IUSBSE image collection; reproduced with permission).

The second chairman of IUSBSE was Michael Lee (professor of biomedical engineering at Dalhousie University, Canada) (Fig. 2). He served from 2000 to 2004 and was assisted by Professor John Ramshaw (Australia), who was the secretary of IUSBSE. The key role of the secretary was to keep the official records of IUSBSE, mainly the minutes of the annual meetings, and to ensure that IUSBSE followed its policies.

The selection of the venues for the next WBC was usually made 8 years in advance. Thus, during WBC2000 in Hawaii, Amsterdam was selected as the venue for WBC2008.

#### Chairman: Young Ha Kim

**Fig. 3.**
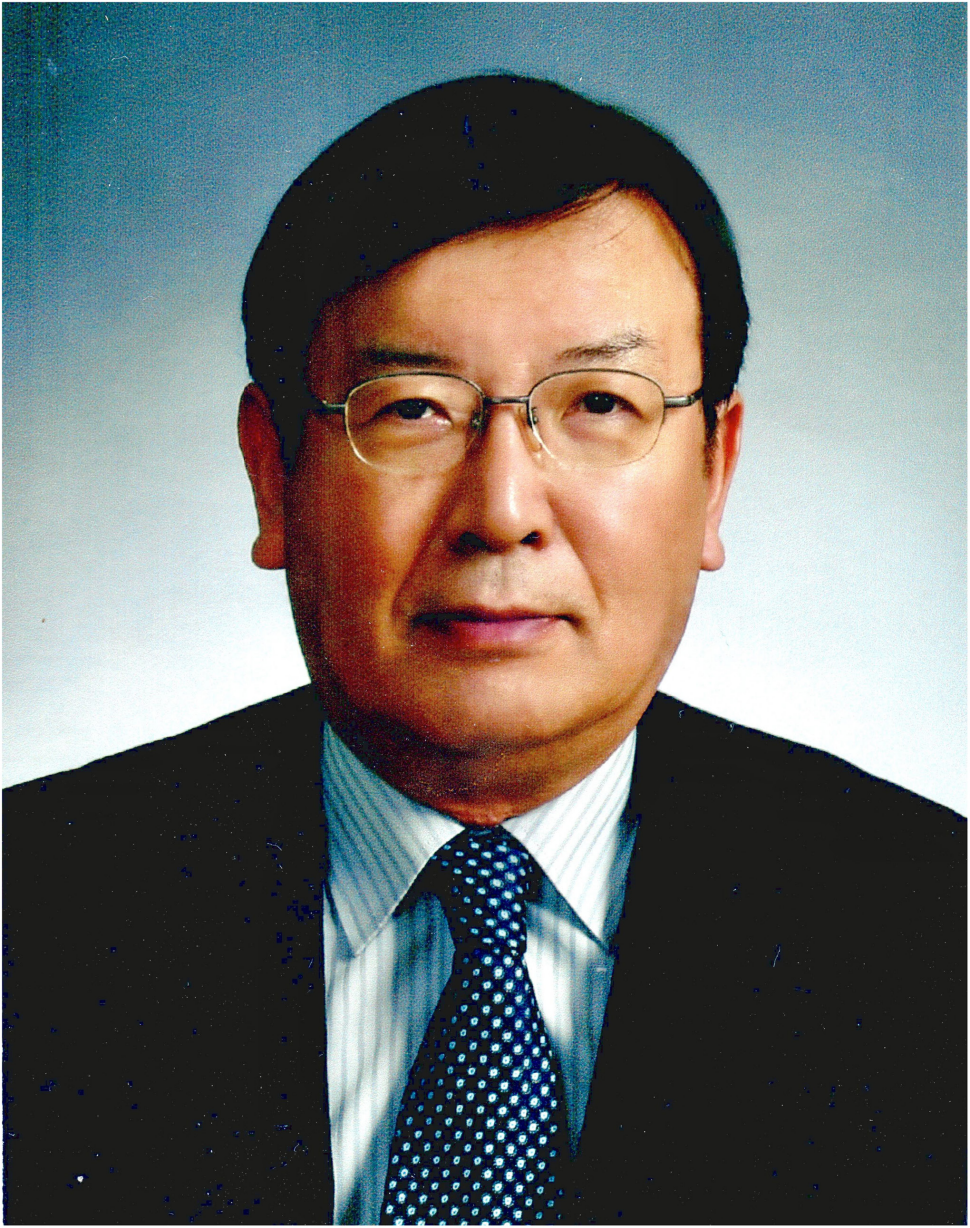
Dr Young Ha Kim, chairman of IUSBSE from 2004 to 2008 (photo © Yong Ha Kim; reproduced with permission).

Professor Young Ha Kim served as the third chairman of IUSBSE from 2004 to 2008 (Fig. 3). Dr John Ramshaw continued to serve as secretary for a second term from 2004 to 2008. Professor Young Ha Kim worked in the Republic of Korea at the Laboratory of Polymer Chemistry, Korea Institute of Science and Technology. He had been the Korean delegate to the International Liaison Committee since 1989. In 2008, Professor Ramshaw reached the end of his term and had to step down. Professor Hasan Uludag was elected as the new secretary for the period 2008 to 2012. Professor Hasan Uludag is a biomedical engineer from the University of Alberta, Canada. The years of Professor Young Ha Kim’s leadership have been characterized by rapid growth in the field of biomaterials. This can be illustrated by comparing the 7th WBC in Sydney, Australia (2004), with the 9th WBC in Chengdu, China (2012) (Table 1).

**Table 1.** Growth of WBC over the period from 2004 to 2012

World Biomaterials Congress	Oral presentations	Attendees
Sydney (Australia) 2004	748	1,700
Chengdu (China) 2012	976	3,500

WBC, World Biomaterials Congress

Under the leadership of Young Ha Kim, IUSBSE had become an established organization with a proven track record in managing the selection of WBC venues and the approval of new Fellows.

During WBC2004, IUSBSE awarded the honor of hosting a WBC to the Chinese Society for Biomaterials (CSBM) and selected Chengdu, China, as the site for WBC2012.

Another important event during WBC2004 was the official launch of the International College of Fellows (ICF). As mentioned earlier, FBSEs are intended to serve as role models for younger scientists and to actively contribute to the advancement of the field of biomaterials science. The first chairman of the ICF was Professor Joachim Kohn, director of the New Jersey Center for Biomaterials (USA) and distinguished professor at Rutgers University (USA). Joachim Kohn and Young Ha Kim worked closely together to establish the ICF as an independent organization under the umbrella of IUSBSE. These efforts culminated in the first “Fellows Debate” during WBC2008 in Amsterdam. Led by Professor David Williams, one team of scientists argued for the necessity of animal experimentation, while another team argued against the practice. This debate became one of the most attended sessions at WBC2008 and started the tradition of “Special Fellows Debates” at all subsequent WBCs as well as most meetings of the ESB and the SFB-USA.

During WBC2008 in Amsterdam, IUSBSE selected Montreal, Canada, as the venue for WBC2016 and awarded the honor of organizing WBC2016 to the Canadian Biomaterials Society (CBS).

#### Chairman: Nicholas Peppas, 2008 to 2016

**Fig. 4.**
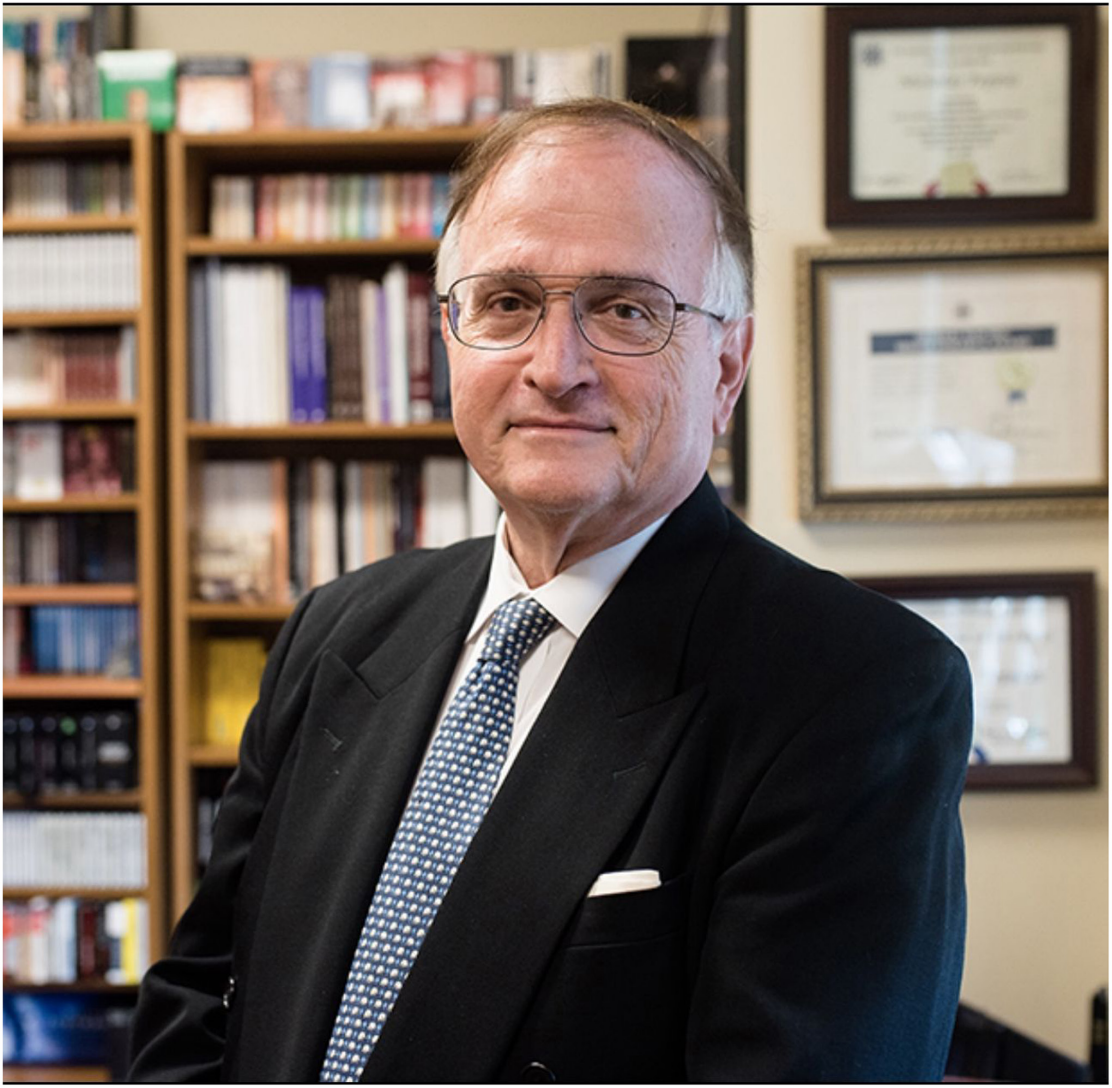
Professor Nicholas Peppas, chairman of IUSBSE from 2008 to 2012 and president of IUSBSE from 2012 to 2016 (photo © Nicholas Peppas; reproduced with permission).

Professor Nicholas Peppas is one of the most prominent biomaterials scientists at the University of Texas at Austin (USA) (Fig. 4). He has excellent working relationships with leading biomaterials scientists around the world. He was an excellent candidate to lead IUSBSE and brought a high level of passion and energy to his position as the chair of IUSBSE. He also had plans to strengthen the internal structure of IUSBSE and explore ways to expand the role of IUSBSE beyond the cautious approach of Andreas von Recum. He was elected at the IUSBSE meeting in Amsterdam in 2008. During his first term from 2008 to 2012, Professor Peppas was assisted as IUSBSE secretary by Dr Hasan Uludag of the University of Alberta in Canada.

When Professor Peppas took office in 2008, he noticed that there was no well-defined procedure for the election of the IUSBSE chair and secretary. Therefore, together with the IUSBSE delegates, he envisioned a new model for the governance of IUSBSE. Dr Peppas proposed to the delegates to change the role of the chairman of IUSBSE to the president of IUSBSE. More importantly, the chairman of IUSBSE has always been elected from among the delegates representing the member societies. According to the governance model of Professor Peppas, the president and the secretary form the “Board of Directors”. The president and the secretary need not be delegates and do not vote in the election of the new president. Any person may be nominated by any IUSBSE delegate for the position of president or secretary. This opens up the talent pool of potential nominees to a wide range of candidates. All delegates then elect the president and secretary by majority vote.

This new process was adopted in 2012 and used for the first time to reelect Professor Peppas to a second term ending in 2016. During his second term, Professor Peppas was assisted by Dr Keith McLean of the Commonwealth Scientific and Industrial Research Organisation (CSIRO) in Australia, who served as IUSBSE secretary.

The world of biomaterials has changed considerably since 1996. While initially the largest group of biomaterials scientists at the early WBCs was from the USA, the participation of Asian scientists (especially Chinese scientists) had increased substantially. The center of the global biomaterials community was shifting from the USA and Europe to Asia. In addition, Internet and communication technologies were beginning to impact the way that scientists could interact with each other.

WBC2012 in Chengdu, China, was a landmark event under the leadership of Professor Xingdong Zhang. This congress had received more than 3,000 abstracts from 57 countries and regions. This congress organized 8 plenary lectures, 1 special public lecture, 87 keynote symposia, 976 oral presentations, and 1,689 poster presentations. The Chinese Society for Biomaterials (CSBM) had created an excellent atmosphere and unparalleled support for all participants.

The ICF organized a debate on the following topic: Can we regrow a human arm? Led by Professor Joachim Kohn, one team of scientists argued for the idea of regrowing whole human limbs, while another team pointed out the difficulties and potential failures of this concept.

At the WBC2012 meeting, IUSBSE selected Glasgow (United Kingdom) as the venue for WBC2020. In addition, IUSBSE had accepted 6 new member societies since 1992, bringing the total number of IUSBSE members to 10 (Table 2).

**Table 2.** Current IUSBSE member societies

Society name
Australasian Society for Biomaterials and Tissue Engineering (ASBTE)
Canadian Biomaterials Society/Société Canadienne des Biomatériaux (CBS/SCB)
Chinese Society for Biomaterials (CSBM)
Chinese Taipei Society for Biomaterials and Controlled Release
European Society for Biomaterials (ESB)
Japanese Society for Biomaterials (JSB)
Korean Society for Biomaterials (KSBM)
Latin American Society for Biomaterials and Artificial Organs (SLABO)
Society for Biomaterials (USA)
Society for Biomaterials and Artificial Organs (India)

#### President Xingdong Zhang, 2016 to 2020

**Fig. 5.**
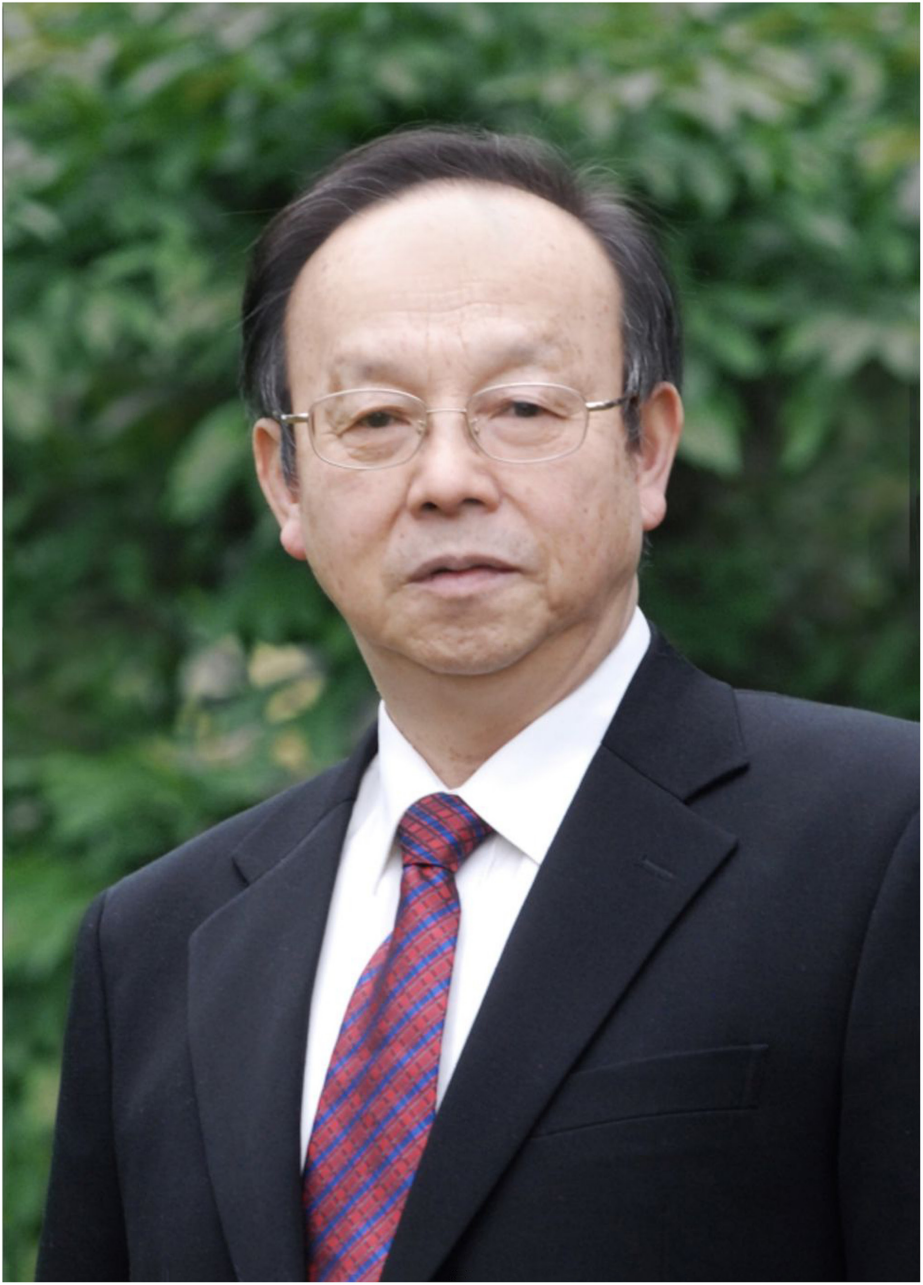
Professor Xingdong Zhang, IUSBSE president from 2016 to 2020 (IUSBSE image collection; reproduced with permission).

Following the new guidelines, Professor Xingdong Zhang was elected as president for the term from 2016 to 2020 (Fig. 5). Professor Keith McLean was reelected as IUSBSE secretary. In the ICF, Professor Joachim Kohn was reelected for another term as chairman of the College of Fellows. Professor Kohn had served as chairman of the College since 2004 and announced that this would be his last term.

During WBC2016, IUSBSE selected Daegu (Republic of Korea) as the venue for WBC2024 and awarded the honor of hosting WBC2024 to the Korean Society for Biomaterials (KSBM). Professor Ki Dong Park served as the chairman of the WBC2024 Organizing Committee.

One of the most impactful actions of Professor Zhang was the organization of a consensus conference in Chengdu (June 2018) to provide updated definitions of biomaterials for the 21st century. This was the first time that IUSBSE supported a consensus conference, providing the global biomaterials community with a set of official definitions for many of the scientific terms used in biomaterials research. Since the consensus conference was held under the auspices of IUSBSE, it was expected that member societies would endorse the book for use by their members.

In December 2019, at the Annual General Meeting of IUSBSE, Professor Joachim Kohn was elected as the next president of IUSBSE. Professor Mario Barbosa was elected as IUSBSE secretary. The official term of the newly elected “Board of Directors” was set to start at the end of the WBC2020.

Everyone was looking forward to WBC2020 in Glasgow (United Kingdom); scientists from all over the world had made travel arrangements and paid the early-bird registration fees. That was when the COVID pandemic began. With all of the critical players in Europe and the USA, Professor Kohn (in his role as president-elect) became the key person to organize the IUSBSE response to the pandemic. Most importantly, in February 2020, it became clear that WBC2020 would not take place as a “face-to-face” meeting as planned. The meeting organizers and IUSBSE had just under 2 months to turn WBC2020 into a virtual meeting. In retrospect, we know that the pandemic has dramatically accelerated the technologies needed for virtual meetings. Today, it is easy to set up a large virtual meeting. In 2020, the technology was still in its infancy.

Despite the difficulties, WBC2020 was as successful as a virtual meeting could be. The web interface was well designed. Video and audio quality was satisfactory for most participants. Overall participation, as measured by people logging into the WBC website, was high. This first virtual WBC lacked the excitement of meeting friends and colleagues in person, and its overall impact was probably diminished. However, the professional conference organizer, the local organizing committee, and the IUSBSE delegates made a heroic effort to provide as much value as possible to the thousands of biomaterials scientists who attended WBC2020 remotely. Due to the inability to travel in May 2020, IUSBSE did not hold its Annual General Meeting and no decision was made regarding the selection of a venue for WBC2028.

#### President Joachim Kohn, 2020 to 2028

**Fig. 6.**
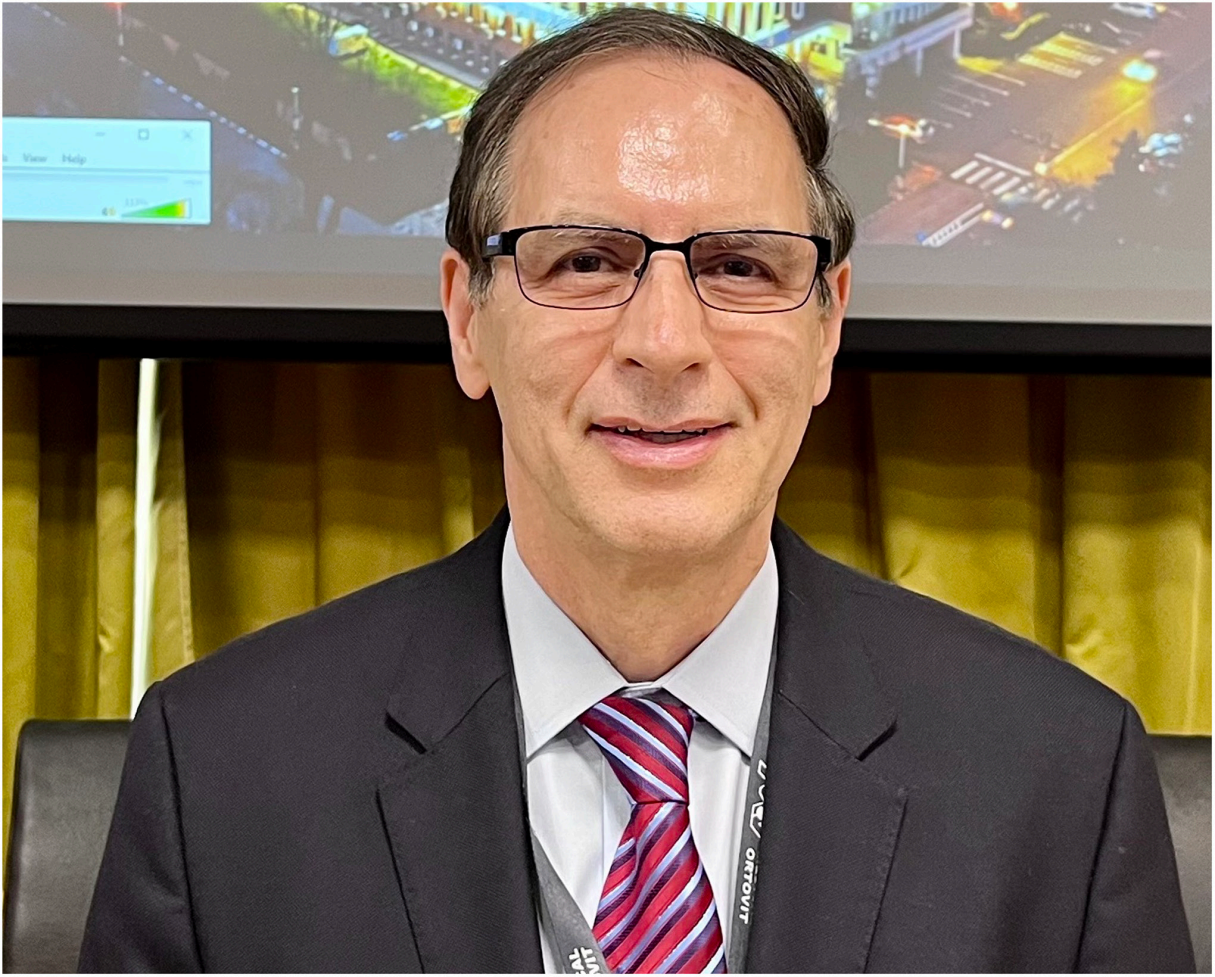
Distinguished Professor Emeritus Joachim Kohn, IUSBSE president from 2020 to 2028 (photo © Joachim Kohn; reproduced with permission).

At the closing session of the virtual WBC2020, Professors Mario Barbosa (University of Porto, Portugal) and Professor Joachim Kohn (Distinguished Professor Emeritus, Rutgers University, USA) (Fig. 6) began their terms as secretary and president, respectively.

During 2020, the pandemic still paralyzed travel and prevented face-to-face meetings. However, the IUSBSE Guidelines were designed for face-to-face meetings and required communication by mail and face-to-face meetings to conduct any official business. Therefore, one of the first actions was to modernize the IUSBSE Guidelines. The updated policies now allow for email communication and voting during virtual meetings. In addition, instead of a single meeting per year, IUSBSE has scheduled 12 delegate meetings between December 2020 and December 2023. During these meetings, a memorandum of understanding with TERMIS was approved. This was an important step forward to develop a closer working relationship between IUSBSE and TERMIS. IUSBSE also agreed on a new procedure for the selection of future WBC venues. This reform was much needed. In previous years, no specific instructions were given to potential applicants on how to prepare a proposal for the WBC and no information was available on how these documents would be evaluated.

The new selection process provides detailed instructions on how to prepare the proposal document and detailed information on how IUSBSE will evaluate the proposals. This new system was used in the selection of the site for WBC2028. IUSBSE awarded the honor of hosting WBC2028 to the SFB-USA. WBC2028 will take place in Washington, DC, USA. Funding for IUSBSE was introduced, which will come from a small surcharge on the registration fee for future WBCs and will be used to support the administration and activities of IUSBSE.

In preparation for WBC2024, IUSBSE elected the president and secretary for the term 2024 to 2028. Professor Mario Barbosa wished to step down and was replaced by Professor Marc Bohner (RMS Institute, Switzerland). Professor Kohn was reelected and will continue as IUSBSE president until the end of WBC2028. As we prepare to meet in Daegu, Korea, for WBC2024, IUSBSE is a strong and vibrant organization that is now well positioned to support the needs of the global biomaterials community. IUSBSE will continue to support international collaboration among its member societies.

Beyond WBC2024, the next important task of President Kohn and the IUSBSE delegates is to select the venue for WBC2032.

## Conclusion

The 12th WBC, held in Daegu, Republic of Korea, from 2024 May 26 to 31 and hosted by the Korean Society for Biomaterials (KSBM), was a meaningful, consequential, and successful event that advanced the understanding and application of biomaterials for human health. As a major international gathering following the coronavirus disease (COVID-19) pandemic, WBC2024 attracted in-person participants and organizers from a wide range of countries, reflecting the global nature of biomaterials research.

The organizers and symposia featured major representatives not only from IUSBSE’s 10 member societies but also from many countries without formal affiliation to IUSBSE. WBC2024 was a large event, with more than 4,000 participants from across the world, representing a substantial gathering of scientists, researchers, academics, and industry professionals in the biomaterials field.

Its success was due to many factors, including global recognition and prestige, a comprehensive scientific program, strong support from the local host, a strategic location, opportunities for networking and collaboration, industry engagement, and excellent organization. The worldwide contributions of the biomaterials community, including strong support from the IUSBSE, also played a key role.

The WBC2024 Organizing Committee and the IUSBSE delegates sincerely thank all IUSBSE presidents for their lifetime contributions to the society and to scientific advancement. This historical overview of the IUSBSE is intended to help society members understand the organization’s role and recognize the lifetime efforts of all its presidents in advancing biomaterials research and commercialization for improved human healthcare and beyond.

